# There Is No News Like Bad News: Women Are More Remembering and Stress Reactive after Reading Real Negative News than Men

**DOI:** 10.1371/journal.pone.0047189

**Published:** 2012-10-10

**Authors:** Marie-France Marin, Julie-Katia Morin-Major, Tania E. Schramek, Annick Beaupré, Andrea Perna, Robert-Paul Juster, Sonia J. Lupien

**Affiliations:** 1 Centre for Studies on Human Stress, Mental Health Research Centre Fernand-Seguin, Louis-H. Lafontaine Hospital, Montreal, Quebec, Canada; 2 Department of Physiology, Faculty of Medicine, Université de Montréal, Montreal, Quebec, Canada; 3 Department of Neurology and Neurosurgery, Faculty of Medicine, McGill University, Montreal, Quebec, Canada; 4 Department of Psychiatry, Faculty of Medicine, Université de Montréal, Montreal, Quebec, Canada; Wayne State University, United States of America

## Abstract

With the advent of specialized television channels offering 24-hour coverage, Internet and smart phones, the possibility to be constantly in contact with the media has increased dramatically in the last decades. Despite this higher access to knowledge, the impact media exposure has on healthy individuals remains poorly studied. Given that most information conveyed in the media is negative and that upon perception of threat, the brain activates the stress system, which leads to cortisol secretion, we decided to determine how healthy individuals react to media information. Accordingly, we investigated whether reading real negative news (1) is physiologically stressful, (2) modulates one’s propensity to be stress reactive to a subsequent stressor and (3) modulates remembrance for these news. Sixty participants (30 women, 30 men) were randomly assigned to either twenty-four real neutral news excerpts or to twenty-four real negative excerpts for 10 minutes. They were then all exposed to a well-validated psychosocial stressor, the Trier Social Stress Test (TSST), which consists of an anticipation phase of 10 minutes and a test phase of 10 minutes. A total of eight salivary cortisol samples were collected, at 10-minutes intervals, throughout the experimental procedure. One day later, a free recall of the news was performed. Results showed that although reading negative news did not lead to change in cortisol levels (*p*>0.05), it led to a significant increase in cortisol to a subsequent stressor in women only (*p*<0.001). Also, women in the negative news condition experienced better memory for these news excerpts compared to men (*p*<0.01). These results suggest a potential mechanism by which media exposure could increase stress reactivity and memory for negative news in women.

## Introduction

Mass media occupies an important place in Westernized societies. Over last decades, industrialized and even developing nations have witnessed the birth of the Internet, specialized news television (TV) channels offering 24-hour coverage, and the advent of smartphones as part of the communication revolution. Considering the potentially threatening nature of the news, it is worth asking how mass media exposition impacts healthy individuals and whether it is stressful.

When the brain perceives a threat, be it real or implicit, the sympathetic-adrenal-medullary (SAM) axis and the hypothalamic-pituitary-adrenal (HPA) axis are both activated. This leads to the secretion of the stress hormones; respectively, catecholamines and glucocorticoids (cortisol in humans). The latter is particularly interesting since it is a steroid that rapidly crosses the blood-brain-barrier and that binds to receptors in different brain regions, notably the hippocampus, the amygdala and the prefrontal cortex [1]. Not surprisingly, there is a strong link between stress hormones and different memory processes. More particularly, stress hormones have been shown to promote memory consolidation [2], [3], the process by which newly learned information that are first unstable in the short-term memory system stabilize in the long-term memory system. Interestingly, this modulation of memory consolidation by stress hormones seems to be especially pronounced for emotional material [4]–[7].

To date, very few studies have assessed the role of mass media as a stressor as well as its impact on reactivity to other stressors in adults. Among those that have, most have been performed using psychological measures in order to determine whether exposure to mass media related to terrorist events increases vulnerability to report different stress symptoms and to develop post-traumatic stress disorder (PTSD). First, as an immediate reaction, it has been shown that the frequency of television watching related to the 9/11 terrorist events was associated with substantial psychological stress reactions among TV watchers when measured in the days following the traumatic event [8]. Second, another study related to the 9/11 attacks reported that the number of hours of TV coverage watched by individuals was correlated with measures of psychological distress and prevalence of probable PTSD [9]. Interestingly, these associations were greater in women when compared to men. Given that women are generally more at risk of developing PTSD following trauma exposure [10], this sexual dimorphism in reactivity to the media suggest that sex differences might be a key variable to take into account when studying physiological and/or psychological reactivity to mass media.

In contrast to psychological measures, the physiological and cognitive effects of everyday news on healthy individuals have not been investigated. To date, only one study measured cortisol levels before and after exposure to real news excerpts and reported no changes in cortisol levels [11]. However, in this study, the news excerpt was the first 30 minutes of a local news program, including weather, sports events and news (crimes and politics). Given that the news excerpt presented to participants comprised both neutral and negative events, it was not possible to dissociate the impact of neutral *versus* negative news on cortisol reactivity in the participants. As well, we know of no study that has assessed whether exposure to real negative news is associated with concomitant modulation of stress reactivity and long-term recall of the news.

In accordance with this lacuna, the goal of the present study was to assess whether exposure to real negative news is (1) physiologically stressful, (2) whether it can modulate one’s stress reactivity to a subsequent validated stressor and (3) whether it can impact the long-term retention for these news in healthy individuals.

## Methods

### Ethics Statement

This study was approved by the ethics committees of the Douglas Mental Health University Institute and the Fernand-Seguin Research Center of Louis-H. Lafontaine Hospital. All participants signed an informed consent before starting the experiment.

### Participants

Sixty healthy francophone men and women between the ages of 18 to 35 years old took part in this study. Recruitment was performed online via advertisements posted on university websites and some general websites. All prospective participants were screened over the phone to make sure they did not suffer from any psychological and/or physical illnesses. All participants were non-smokers and did not take any medication. Participants were either exposed to neutral or negative news (between-subject design). There were 15 men and 15 women in each news condition. Four participants (two men and two women) had extreme baseline cortisol values (+2 SD above the mean). For this reason, these participants were taken out of all analyses. The final sample size was thus 56 participants (14 men and 14 women in each condition). From this sample, 14 women were using oral contraceptives (3 from the emotional condition and 11 from the neutral condition). Five women (4 from the emotional condition and 1 from the neutral condition) were tested in the follicular phase of their menstrual cycle whereas five women (4 from the emotional condition and 1 from the neutral condition) were tested in the luteal phase of their cycle. Information about the cycle was missing for 4 participants (3 from the emotional condition and 1 from the neutral condition).

### Task Design

In order to control for bisensory augmentation effects of news exposure on memory performance [12], [13], all news excerpts were presented visually as written news. News were collected from the two most read francophone newspapers in the region of Montreal; namely, the Journal de Montréal® and La Presse®. Each news excerpts’ condition (negative vs. neutral) was created by extracting twelve neutral news and twelve negative news from each newspaper. Each news stimuli consisted of the title and a short excerpt (the first lines of the article summarizing the event). The same individual (M.F.M.) selected all the excerpts used for this study. To ensure that this researcher’s judgment about valence (neutral vs. negative) was valid, a task validation analysis was performed (see task validation in results section).

In order to ensure that the news were relatively equal in timing since occurrence, the task was constantly updated so that the participants were exposed to twenty-four news excerpts that were all published within the frame of one month (for each condition, three news excerpts per journal per week). This constant updating of the task resulted in a total of seven different stimuli sets for each condition. The task was programmed using E-Prime^©^ and lasted ten minutes. For each group, news were presented in a randomized order where each excerpt stayed on the screen for twenty-five seconds. Participants were told that recent news from the media would be displayed on the screen and that their task was to carefully read them in silence. Participants were not informed about the later memory test (incidental learning).

### Psychological Stressor

In order to expose participants to a psychosocial stressor, we used the Trier Social Stress Test (TSST) [14]. The TSST protocol used in the present study was slightly different from the original procedure [14]. In the original version of the TSST, participants are in the same room as the judges (‘Panel-in condition’) whereas in our study, the judges were behind a false mirror (‘Panel-out condition’). The reason why we decided to use the ‘Panel-out’ variant in the present study is that the students who acted as judges in our experiment were often younger than the participants, and age of the judges has been suggested to have a significant impact on the amount of socio-evaluative threat induced by the TSST in previous studies. Therefore, in order to avoid the confounding effect of socio-evaluative threat induced by the age of the judges, we limited to a minimum the contact between the participants and the judges. We have used this ‘Panel-out’ version in many of our studies [15]–[18]. Andrews and colleagues have reported no differences between the ‘Panel-in condition’ and the ‘Panel-out condition’ in men [15] whereas Wadiwalla and colleagues have reported higher cortisol reactivity in the ‘Panel-in condition’ compared to the ‘Panel-out condition’ in women [16]. Beside the location of the panel in the modified version of the TSST, all conditions related to this procedure were the same as in the original procedure.

In summary, the task involves an anticipation phase (10 minutes) and a test phase (10 minutes), which is divided into a mock job interview (5 minutes) followed by mental arithmetic (5 minutes). Throughout their performance, participants are facing a false mirror and a camera. Behind this mirror, two confederates who act as judges and pretend to be experts in behavioural analysis observe the participants and communicate with them via an intercommunication system. All participants were introduced to the judges between the anticipation and test phases.

### Procedure

Participants came to the laboratory for a single session that took place between 12:30PM and 17:00PM to control for the circadian rhythm of cortisol. Upon arrival, participants had to rest for fifteen minutes. Upon informed consent and this habituation phase, participants provided a first saliva sample where they were asked to fill a small plastic vial with 1 ml of pure saliva (i.e., passive drool). All participants were then randomly assigned to either the neutral or negative condition for the newspaper task, which lasted for ten minutes. Immediately following this procedure as well as ten minutes after, saliva samples were collected again (samples 2 and 3). After completing sample 3, the experimenter instructed the participants about the TSST and let them prepare their mock job interview speech. They were then brought to the room and introduced to the judges and to the experimental setting before taking another saliva sample (sample 4). They then had to do the verbal (5 minutes) and the mental arithmetic (5 minutes) tasks. Following this, they were brought back to the initial room. Immediately after this, participants were asked to provide another saliva sample (sample 6) and to rate the stressfulness of the TSST task on a scale from 1 to 10 (1 being not stressful and 10 being very stressful). Ten and twenty minutes later, participants provided saliva samples (7 and 8). Once this was completed, a phone appointment was fixed with the participant for the following day and they were then allowed to return home.

One day later, the experimenter called back the participants at their convenience. Participants expected the phone call, but were told that the goal was to discuss further the objectives of the study and to ask some follow-up questions. During this call, participants were then asked to recall as many news as possible from the newspaper task they were exposed to the day before. They were encouraged to give as many details as possible. The experimenter transcribed everything that was said by the participant and two raters scored the answers. A score of 1 was attributed for every excerpt recalled by the participant. It was necessary for the two raters to be able to identify the exact news excerpt that the participant was talking about in order to allocate a point. A total memory score was computed for each participant.

Following the memory test, the experimenter read each news excerpt to which the participant was exposed the day before. Using scales from 1 to 5, the participant had to rate the emotionality of each excerpt (1 being very neutral and 5 being very emotional) as well as the extent to which they felt concerned by each of them (1 being not concerned at all and 5 being very concerned).

### Salivary Cortisol Assays

Saliva samples were stored at −20°C until time of cortisol concentration determination. Analyses were performed at the Centre for Studies on Human Stress (www.humanstress.ca) using a high sensitivity Enzyme immune assay kit from Salimetrics State College, PA, catalogue number 1-3102. The range of detection for this assay is 0.012–3 ug/dL. All samples were assayed in duplicates. The intra-assay coefficient of variation (CV) ranged between 3.35% and 3.65% and the inter-assay CV ranged between 3.75% and 6.41%.

### Data Analysis

#### Treatment of cortisol data

Cortisol values followed a normal distribution and for this reason, raw data of cortisol were used for all analyses.

#### Emotional rating of the news

As mentioned above, the participants rated each news excerpt on two components: (1) emotionality and (2) the extent to which they felt concerned by it. Thus, for each participant, we computed an average of the 24 scores (one per excerpt) obtained for each of these components (‘emotionality’ and ‘concerned’). Because the stimuli had to be regularly updated in order to ensure that the news to which each participant was exposed occurred within the last month, this resulted in a total of seven different sets of stimuli. In order to determine whether all these sets were equivalent in terms of emotionality and the extent to which participant felt concerned by them, and to allow collapsing across the different sets of stimuli, multivariate analyses were performed for each Condition (neutral, negative) on these two dependent variables (emotionality and concerned) with sets of stimuli (1 to 7) as the independent variable.

#### Task validation analysis

It was then necessary to validate the newspaper task that we used. To do so, we had to make sure that the news we picked for the negative condition were more emotional and negative to people than the selected neutral news. Thus, 2-way ANOVAs were conducted on the ‘emotionality’ and the ‘concerned’ scores with Sex (men vs. women) and Condition (neutral vs. emotional) as the between subject factors.

#### Main analyses

In order to determine whether reading negative news was stressful and whether it modulates stress reactivity to another stressor, two 3-way mixed design ANOVA were performed with Time, Sex and Condition. In order to determine whether the subjective stress perception of the TSST differed in the various conditions, we conducted a 2-way mixed design ANOVA with Condition and Sex on the score given by the participants after completion of the TSST (scale from 1 to 10). To investigate the impact on memory, a 2-way between-subjects ANOVA was ran with Condition and Sex. For each analysis, the average score for ‘emotionality’ and ‘concerned’ were entered as covariates. Greenhouse-Geisser values were used when the assumption of sphericity was violated [19].

#### Supplementary analyses

Given that women were not necessarily tested in the same menstrual cycle phase and that some of them were taking oral contraceptives, we added a covariate ‘cycle’ in all analyses pertaining to women. The factor was divided in three levels: follicular phase, luteal phase or contraceptive pill.

## Results

### Sets of Stimuli

Multivariate analyses performed on the ‘emotionality’ and the ‘concerned’ scores revealed no effect of Sets of stimuli for each Condition (neutral, negative), *F*_s_(6, 21) <1.276, *p_s_* >0.289. Therefore, for each condition, data were collapsed across the seven stimuli sets.

### Task Validation

Multivariate analyses revealed a main effect of Condition on the ‘emotionality’, *F*(1, 52) = 7.308, *p*<0.001, and the ‘concerned’ scores, *F*(1, 52) = 1.811 *p* = 0.026, where the negative news task received a higher score than the neutral news task on both of these measures. Moreover, a main effect of Sex on the ‘emotionality’ score was revealed, *F*(1, 52) = 2.212, *p* = 0.019, where men rated news as being more emotional than women. In both cases, the interaction between Condition and Sex failed to reach significance, *F*_s_(1, 52)<1.084, *p_s_*>0.082. To rule out the contribution of these factors (emotionality and concerned), they were used as covariates in all subsequent analyses.

### Main Analyses

**Figure 1:**
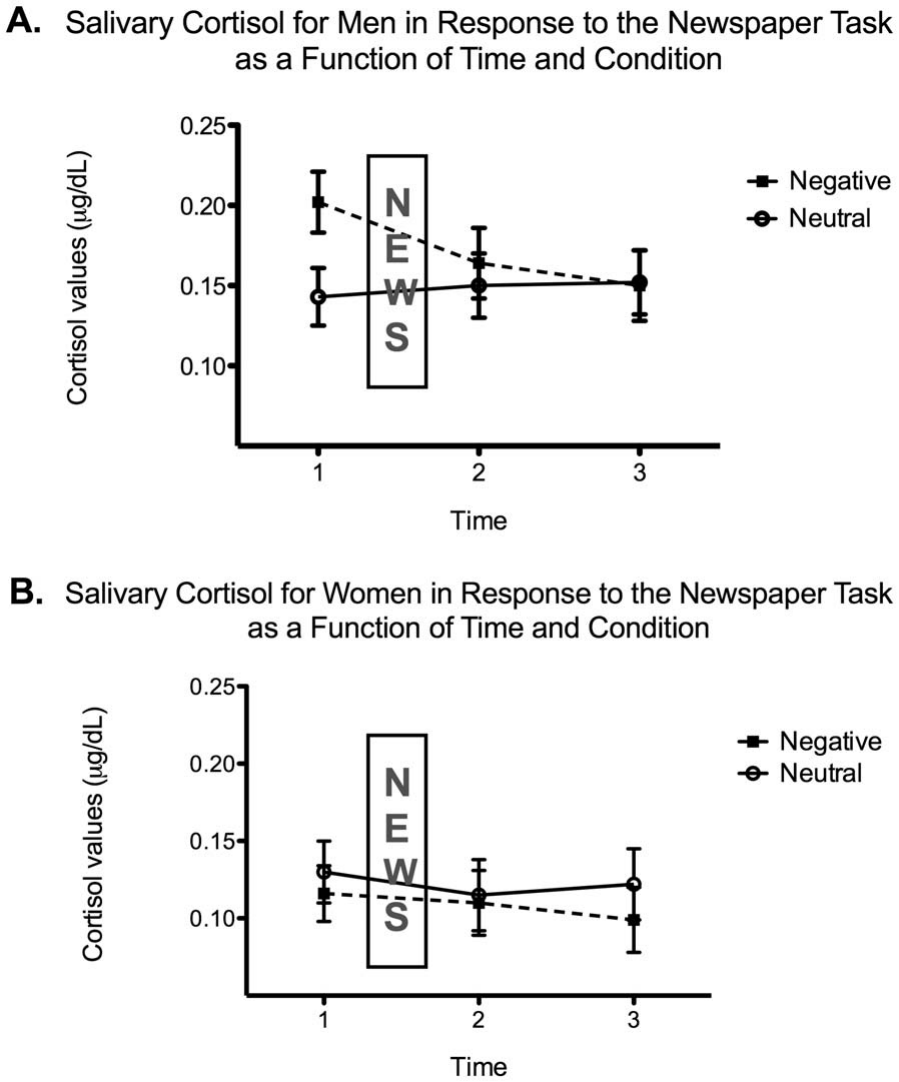
Mean salivary cortisol levels in response to the newspaper task as a function of Time (1– baseline, 2– immediately following the newspaper task, 3– ten minutes after the end of the newspaper task) and Condition (neutral, negative) for men (panel A) and women (panel B). Adjusted means as a function of ‘emotionality’ and ‘concerned’ are presented. The error bars represent the standard error of the mean.

With regards to the analysis testing whether reading negative news was physiologically stressful, a 3-way mixed ANOVA with Time (time 1 baseline, time 2 post-newspaper, time 3 ten minutes post-newspaper), Sex (men vs. women) and Condition (neutral vs. negative) was conducted on salivary cortisol values (see Figure 1). The analysis only revealed a significant main effect of Sex, *F*(1, 50) = 5.893, *p* = 0.019, with men having overall higher cortisol levels than women.

**Figure 2:**
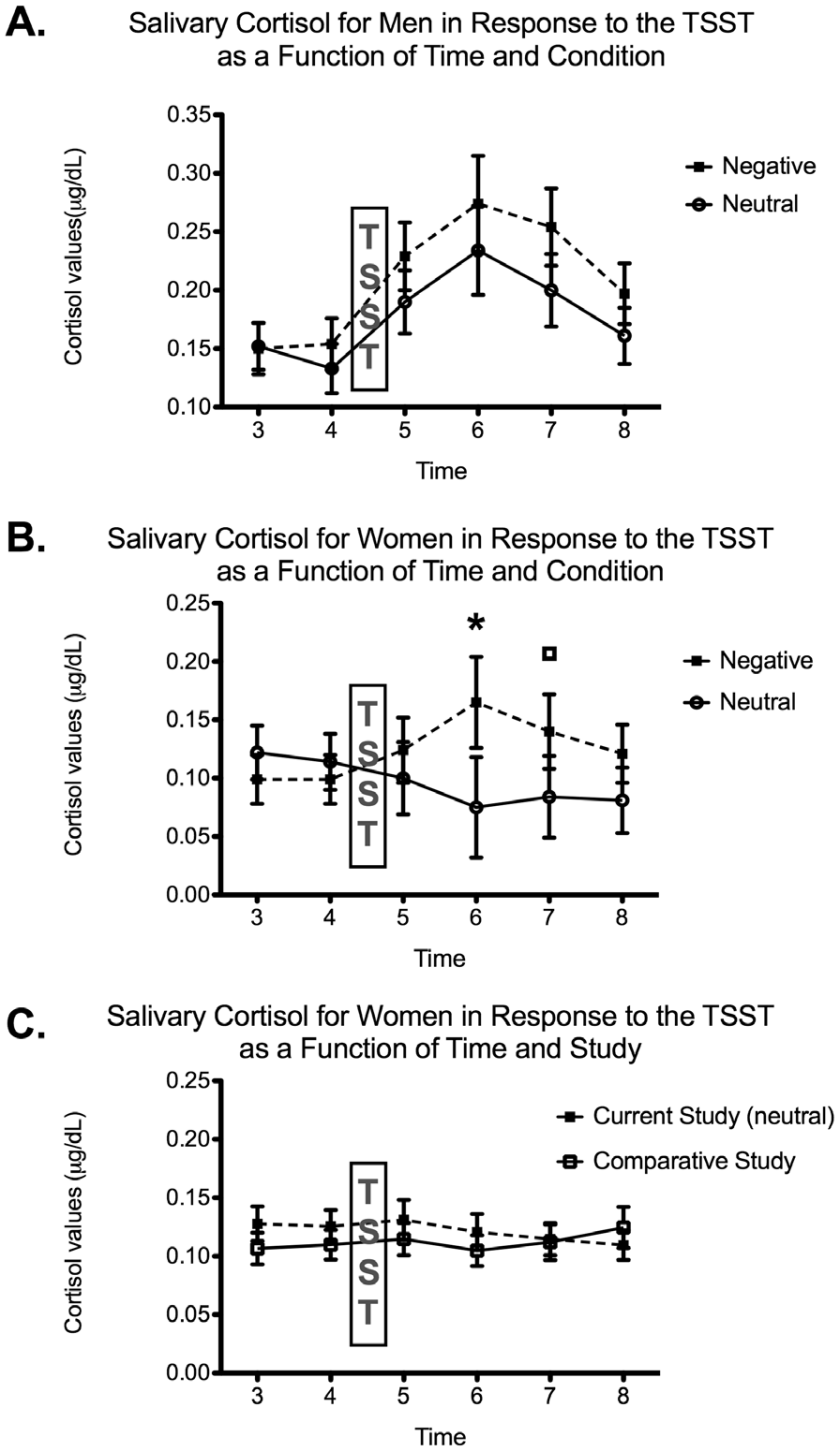
Mean salivary cortisol levels in response to the Trier Social Stress Test as a function of Time (Time 3– pre-anticipation, Time 4– post-anticipation, Time 5, 6,7 and 8–0, 10, 20 and 30 minutes following the end of the TSST) and Condition (neutral, negative) for men (panel A) and women (panel B). Adjusted means as a function of ‘emotionality’ and ‘concerned’ are presented. The error bars represent the standard error of the mean. The asterisk (*) means p<0.05 and the square (□) stands for trend towards significance (p = 0.072). Panel C depicts mean salivary cortisol levels in women in response to the Trier Social Stress Test as a function of Time (Time 3– pre-anticipation, Time 4– post-anticipation, Time 5, 6,7 and 8–0, 10, 20 and 30 minutes following the end of the TSST) and Study (current study – group of women neutral vs. comparative study – group of women). The raw means are presented. The error bars represent the standard error of the mean.

For the analysis regarding physiological reactivity to a subsequent validated psychosocial stressor, a 3-way mixed ANOVA with Time (time 3 pre-anticipation, time 4 post-anticipation, time 5 post-TSST, time 6, 7 and 8), Sex (men vs. women) and Condition (neutral vs. negative) was conducted on salivary cortisol values. The analysis yielded significant main effects of Time, *F*(1.717, 85.87) = 4.584, *p* = 0.017 and Sex, *F*(1, 50) = 10.301, *p* = 0.002. Moreover, the interactions of Time X Condition and Time X Sex were both significant, all *F*_s_(1.717, 85.87)>3.668, all *p*_s_<0.036. Because of the sex effect, 2-way ANOVAs with Time and Condition were conducted for each sex with ‘emotionality’ and ‘concerned’ as covariates. For men, there was no main effect of Condition, *F*(1, 24) = 0.408, *p*>0.05 nor did it interact with Time, *F*(1.636, 39.259) = 0.433, *p*>0.05 (see Figure 2A). For women, Time was significant, *F*(1.974, 47.377) = 7.193, *p* = 0.002 and it also significantly interacted with Condition, *F*(1.974, 47.377) = 10.715, *p*<0.001. Multivariate analyses with Condition as the independent variable and ‘emotionality’ and ‘concerned’ as covariates revealed that women exposed to negative news had significantly higher cortisol levels than the women exposed to neutral news at time 6, *F*(1, 24) = 5.514, *p* = 0.027 and time 7, *F*(1, 24) 3.545, *p* = 0.072 (see Figure 2B).

With regards to the subjective perception of the TSST (score given on a scale from 1 to 10), a 2-way between-subjects ANOVA with Sex and Condition was performed with the covariates ‘emotionality’ and ‘concerned’. The analysis only revealed a significant main effect of Sex, *F*(1,50) = 4.745, *p* = 0.034, where women reported finding the task more stressful than men.

**Figure 3:**
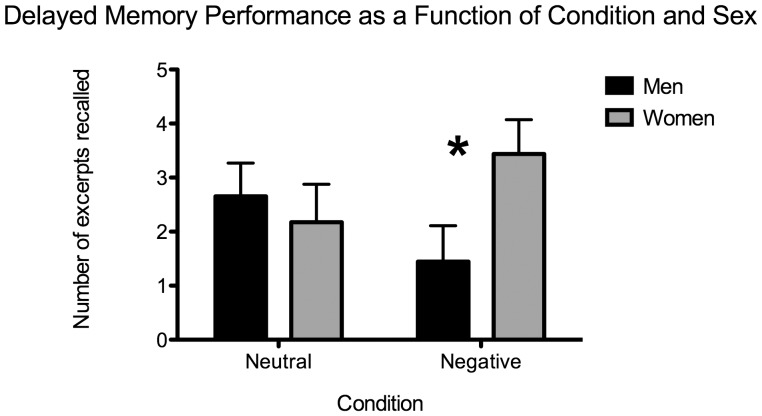
Mean delayed memory performance for the news excerpts as a function of Condition (neutral, negative) and Sex (men, women). Adjusted means as a function of ‘emotionality’ and ‘concerned’ are presented. The error bars represent the standard error of the mean. The asterisk (*) means p<0.05.

With regards to memory performance, a 2-way between-subjects ANOVA with Sex (men vs. women) and Condition (neutral vs. negative) was computed on the total memory score, using the same two covariates than mentioned above. This revealed a significant interaction of Sex X Condition, *F*(1, 50) = 3.818, *p* = 0.056. Since emotional memories are better remembered than neutral ones, one-way ANOVAs were conducted for each Condition (neutral, negative) with Sex as the independent variable and ‘emotionality’ and ‘concerned’ as covariates. For the neutral condition, there was no effect of Sex, *F*(1, 24) = 1.936, *p*>0.05. For the negative news condition, there was a significant main effect of Sex, *F*(1, 24) = 8.103, *p* = 0.009 with women recalling significantly more negative news than men (see Figure 3).

### Supplementary Analyses

In order to determine whether oral contraceptives or the menstrual cycle phase would impact on the results, all analyses pertaining to women (cortisol, subjective stress of the TSST and memory) were re-ran with ‘cycle’ as a covariate. All of these analyses revealed a non-significant effect of the variable ‘cycle’, *p_s_*>0.419.

When looking at Figures 2A and 2B, one could notice that women in the neutral condition seem to not respond to the TSST task. In order to make sure that our group of women in the neutral condition had a response that was comparable to what is observed in other studies using this version of the TSST (‘Panel-out condition’), we compared the raw cortisol data (not adjusted means) of our group of women who were assigned to the neutral condition with a subgroup of women with a similar age range (n = 19) who took part in another study from our laboratory [20]. The saliva samples from the two studies were analyzed using the same enzyme immunoassay method from the Centre for Studies on Human Stress and were thus comparable. The 2-way mixed design ANOVA with Time and Study performed on the raw cortisol data did not yield a main effect of Study nor an interaction of Time X Study, *F*_s_(1, 52)<0.879, *p_s_*>0.497 (see Figure 2C) showing that the low cortisol reactivity observed in women exposed to neutral news was related to the ‘Panel-out’ condition variation of the TSST and not to the effects of news per se.

## Discussion

In the present study, we found that reading neutral or negative news does not lead to a significant increase in cortisol levels in men and women. However, we showed that exposure to real negative news significantly increases physiological reactivity to a subsequent stressor, an effect that is specific to women who have been previously exposed to negative news. Finally, we showed that women who are exposed to stress after reading negative news have greater remembrance for these news.

The fact that newspaper reading did not result in cortisol increase is in line with the study by Ragonesi & Antick [11] whereby no modulation of cortisol levels as a function of news watching was reported. As previously mentioned, this particular study used a segment of local news covering neutral and emotional material (politics, sports, crimes and weather). In the current study, the between-subject design allowed us to compare negative news to neutral ones and the same effect was found.

The important finding of this study is that exposure to negative news significantly increases physiological reactivity to a *subsequent* stressor, an effect that is specific to women and not observed in the group of individuals (men and women) that read neutral news before being exposed to a psychosocial stressor. As shown in Figure 2B, women who were exposed to neutral news had a very low (if any) reactivity to the TSST. This finding is in accordance with previous studies that have reported lower stress reactivity to this particular stress task in women when compared to men [21], [22]. Our study used a false mirror for the stress task (‘Panel-out condition’) so that the participant could not see the judges while he/she was performing the speech and the mathematical task. It has been reported that women are sensitive to this manipulation and show a lower reactivity to the stressor when this manipulation is applied [16], which is not the case for men [15]. We nonetheless decided to use this variant of the TSST (false mirror) since the judges were undergraduate students, who were often younger than the participant being tested. Importantly, our data were compared to another database where the false mirror was also used and no significant difference was found. Therefore, we are confident that our group of women who were in the neutral condition is representative of other studies using the TSST with the ‘Panel-out’ variant. All other things being equal between the two conditions (neutral vs. emotional), this suggests that the higher reactivity we reported in the negative news group is not an artifact created by an abnormal control group (neutral condition) but is rather attributable to the negative news exposition.

At this point, it is not clear why the phenomenon of stress reactivity modulation by previous reading negative news was not observed in men. When visualizing cortisol values for the TSST phase in men, it does demonstrate similar pattern as that observed in women (higher reactivity in men exposed to negative news). Clearly, this remains speculative since the lack of statistical significance does not allow us to make such a statement. Given that men react more to the TSST than women, it is possible that this greater reactivity renders modulation by external agents harder to be attained. Future studies could use a similar design but a different stress task known to affect women more than men, such as the Yale Interpersonal Stressor (YIPS) [23]. That way, men’s reactivity to the stressor would be lower, allowing more space for modulation to take place.

Different mechanisms could be proposed to account for the obtained results. First, previous studies performed in rodents have shown that the HPA axis can habituate to the same, repeated stressor when cumulatively exposed. However, there is a price to pay for this habituation effect. Indeed, Bhatnagar and Dallman [24] have shown that when rodents are exposed to a repeated chronic stressor, this leads to an habituation effect whereby the cortisol response to this stressor is decreased. However, if the rat is exposed to any other new stressor, the capacity of the HPA axis to mount a stress response is preserved and even enhanced. Given that a significant proportion of men and women read the newspapers headlines everyday, it may be possible that even if some negative news excerpts are stressful, chronic exposure to these same types of news leads to a habituation effect. This would explain the absence of significant cortisol reactivity observed in both men and women who were exposed to the negative news condition. However, this habituation to the same chronic stressor (newspaper reading) could lead to increased reactivity to any new stressor as revealed in rodent studies.

The increased reactivity to the TSST that we observed in women who were previously exposed to negative new excerpts suggests that a facilitation of stress reactivity to a new stressor could be at play here. Based on these results, we suggest that exposition to negative news media on a regular basis can have its toll on the capacity of women to more strongly react to other environmental stressors of their daily life.

Another mechanism that could explain the obtained findings relates to the presence of higher ruminative tendencies in women [25]. Ruminating about the bad news that they have just read might have primed women for greater stress reactivity to the TSST, by biasing their perception of the task. However, this seems unlikely given that the subjective perception of the TSST was greater among women, but did not differ as a function of condition. Importantly, this result goes along with the literature suggesting a greater perception of stress by women when compared to men [26].

Alternatively, the ruminative capacities might explain the higher memory performance observed in the group of women who were exposed to the negative news. If negative news exposition leads to more rumination among women, this suggests a greater propensity towards cognitively elaborating and extenuating information that ultimately promotes greater consolidation processes. This increase in memory consolidation would therefore manifest itself in the form of increased memory performance, which was observed in the group of women exposed to negative news. Importantly, our memory results are in line with a recent review that reported higher emotional memory capacities in women [27]; however, the precise mechanisms of this sex difference remains elusive.

The current data could also be approached from an evolutionary perspective. In fact, it has been proposed that men and women’s stress systems have evolved differently by serving distinct purposes [28]. In fact, it has been suggested that the women’s stress system is wired-up to ensure not only their own survival but the one of their offspring as well. This requires a certain degree of empathy, a characteristic that seems more pronounced in women than in men [29]. Given that the majority of the news excerpts involve the capacity to detect threats that are directed to other people and not to the self, it is thus possible that the task had a stronger effect on the physiological stress system of women. Along the same line, this evolutionary-based mechanism could also promote women’s memory for the negative news. In fact, in order to ensure the protection of their offspring, it is primordial for women to remember the potential threats surrounding their environment. Although this evolutionary mechanism is hardly testable, it would be interesting to include measure of empathy with regards to sex differences in reactivity to stress after exposure to negative news in future studies.

In summary, this study demonstrated that exposure to real negative news has the capacity to modulate women’s stress reactivity to a subsequent psychosocial stressor as well as their memory performance for these negative news. Our results underline the importance that mass media may exert on physiological and psychological processes that should be further investigated more broadly in the context of everyday news and following major traumatic events. While our results are specific to women, future studies should investigate various populations as function of, for instance, gender-based, generational, and other socio-cultural factors that modulate individual differences in propensities towards informing themselves and coping with negative news. Given that “there is no news like bad news”, it is essential to understand the societal reactions to negative information that is perpetually transmitted and passively received via popular mediums.

## References

[1] Herman JP, Ostrander MM, Mueller NK, Figueiredo H (2005) Limbic system mechanisms of stress regulation: hypothalamo-pituitary-adrenocortical axis. Prog Neuropsychopharmacol Biol Psychiatry 29: 1201–1213. 16271821 10.1016/j.pnpbp.2005.08.006

[2] Beckner VE, Tucker DM, Delville Y, Mohr DC (2006) Stress Facilitates Consolidation of Verbal Memory for a Film but Does Not Affect Retrieval. Behav Neurosci 120: 518–527. 16768603 10.1037/0735-7044.120.3.518

[3] Schwabe L, Bohringer A, Chatterjee M, Schachinger H (2008) Effects of pre-learning stress on memory for neutral, positive and negative words: Different roles of cortisol and autonomic arousal. Neurobiol Learn Mem 90: 44–53. 18334304 10.1016/j.nlm.2008.02.002

[4] Cahill L, Gorski L, Le K (2003) Enhanced human memory consolidation with post-learning stress: interaction with the degree of arousal at encoding. Learn Mem 10: 270–274. 12888545 10.1101/lm.62403 PMC202317

[5] Buchanan TW, Lovallo WR (2001) Enhanced memory for emotional material following stress-level cortisol treatment in humans. Psychoneuroendocrinology 26: 307–317. 11166493 10.1016/s0306-4530(00)00058-5

[6] Kuhlmann S, Wolf OT (2006) Arousal and Cortisol Interact in Modulating Memory Consolidation in Healthy Young Men. Behav Neurosci 120: 217–223. 16492134 10.1037/0735-7044.120.1.217

[7] Payne JD, Jackson ED, Hoscheidt S, Ryan L, Jacobs WJ, et al (2007) Stress administered prior to encoding impairs neutral but enhances emotional long-term episodic memories. Learn Mem 14: 861–868. 18086830 10.1101/lm.743507 PMC2151024

[8] Schuster MA, Stein BD, Jaycox L, Collins RL, Marshall GN, et al (2001) A national survey of stress reactions after the September 11, 2001, terrorist attacks. N Engl J Med 345: 1507–1512. 11794216 10.1056/NEJM200111153452024

[9] Schlenger WE, Caddell JM, Ebert L, Jordan BK, Rourke KM, et al (2002) Psychological reactions to terrorist attacks: findings from the National Study of Americans’ Reactions to September 11. JAMA 288: 581–588. 12150669 10.1001/jama.288.5.581

[10] Kessler RC, Sonnega A, Bromet E, Hughes M, Nelson CB (1995) Posttraumatic stress disorder in the National Comorbidity Survey. Arch Gen Psychiatry 52: 1048–1060. 7492257 10.1001/archpsyc.1995.03950240066012

[11] Ragonesi AJ, Antick JR (2008) Physiological responses to violence reported in the news. Percept Mot Skills 107: 383–395. 19093600 10.2466/pms.107.2.383-395

[12] Frieske DA, Park DC (1999) Memory for news in young and old adults. Psychol Aging 14: 90–98. 10224634 10.1037//0882-7974.14.1.90

[13] Stine EA, Wingfield A, Myers SD (1990) Age differences in processing information from television news: the effects of bisensory augmentation. J Gerontol 45: P1–8. 2295777 10.1093/geronj/45.1.p1

[14] Kirschbaum C, Pirke KM, Hellhammer DH (1993) The ‘Trier Social Stress Test’–a tool for investigating psychobiological stress responses in a laboratory setting. Neuropsychobiology 28: 76–81. 8255414 10.1159/000119004

[15] Andrews J, Wadiwalla M, Juster RP, Lord C, Lupien SJ, et al (2007) Effects of manipulating the amount of social-evaluative threat on the cortisol stress response in young healthy men. Behav Neurosci 121: 871–876. 17907819 10.1037/0735-7044.121.5.871

[16] Wadiwalla M, Andrews J, Lai B, Buss C, Lupien SJ, et al (2010) Effects of manipulating the amount of social-evaluative threat on the cortisol stress response in young healthy women. Stress 13: 214–220. 20392193 10.3109/10253890903277561

[17] Lupien SJ, Gaudreau S, Tchiteya BM, Maheu F, Sharma S, et al (1997) Stress-induced declarative memory impairment in healthy elderly subjects: relationship to cortisol reactivity. J Clin Endocrinol Metab 82: 2070–2075. 9215274 10.1210/jcem.82.7.4075

[18] Juster RP, Perna A, Marin MF, Sindi S, Lupien SJ (in press) Timing is everything: Anticipatory stress dynamics among cortisol and blood pressure reactivity and recovery in healthy adults. Stress. 10.3109/10253890.2012.661494 22296506

[19] Greenhouse S, Geisser S (1959) On methods in the analysis of profile data. Psychometrika 24: 95–112.

[20] Juster RP, Smith NG, Ouellet E, Sindi S, Lupien SJ (under review) Sexual minority stress and disclosure in relation to psychiatric symptoms, diurnal cortisol, and allostatic load. Psychosom Med. 10.1097/PSY.0b013e3182826881 23362500

[21] Kudielka BM, Hellhammer J, Hellhammer DH, Wolf OT, Pirke KM, et al (1998) Sex differences in endocrine and psychological responses to psychosocial stress in healthy elderly subjects and the impact of a 2-week dehydroepiandrosterone treatment. J Clin Endocrinol Metab 83: 1756–1761. 9589688 10.1210/jcem.83.5.4758

[22] Childs E, Dlugos A, De Wit H (2010) Cardiovascular, hormonal, and emotional responses to the TSST in relation to sex and menstrual cycle phase. Psychophysiology 47: 550–559. 20070572 10.1111/j.1469-8986.2009.00961.x PMC4242596

[23] Stroud LR, Tanofsky-Kraff M, Wilfley DE, Salovey P (2000) The Yale Interpersonal Stressor (YIPS): affective, physiological, and behavioral responses to a novel interpersonal rejection paradigm. Ann Behav Med 22: 204–213. 11126465 10.1007/BF02895115

[24] Bhatnagar S, Dallman M (1998) Neuroanatomical basis for facilitation of hypothalamic-pituitary-adrenal responses to a novel stressor after chronic stress. Neuroscience 84: 1025–1039. 9578393 10.1016/s0306-4522(97)00577-0

[25] Strauss J, Muday T, McNall K, Wong M (1997) Response style theory revisited: Gender differences and stereotypes in rumination and distraction. Sex Roles 36: 771–792.

[26] Kudielka BM, Kirschbaum C (2005) Sex differences in HPA axis responses to stress: a review. Biol Psychol 69: 113–132. 15740829 10.1016/j.biopsycho.2004.11.009

[27] Andreano JM, Cahill L (2009) Sex influences on the neurobiology of learning and memory. Learn Mem 16: 248–266. 19318467 10.1101/lm.918309

[28] Taylor SE, Klein LC, Lewis BP, Gruenewald TL, Gurung RA, et al (2000) Biobehavioral responses to stress in females: tend-and-befriend, not fight-or-flight. Psychol Rev 107: 411–429. 10941275 10.1037/0033-295x.107.3.411

[29] Eisenberg N, Lennon R (1983) Sex differences in empathy and related capacities. Psychol Bull 94: 100–131.

